# The CUSSH programme: supporting cities’ transformational change towards health and sustainability

**DOI:** 10.12688/wellcomeopenres.16678.2

**Published:** 2021-11-09

**Authors:** Michael Davies, Kristine Belesova, Melanie Crane, Joanna Hale, Andy Haines, Emma Hutchinson, Gregor Kiesewetter, Blessing Mberu, Nahid Mohajeri, Susan Michie, James Milner, Gemma Moore, David Osrin, Helen Pineo, Irene Pluchinotta, Aarathi Prasad, Giuseppe Salvia, Phil Symonds, Jonathon Taylor, Catalina Turcu, Ioanna Tsoulou, Nici Zimmermann, Paul Wilkinson

**Affiliations:** 1 Bartlett School of Environment, Energy and Resources, University College London, Institute for Environmental Design and Engineering, London, UK; 2 London School of Hygiene and Tropical Medicine, London, UK; 3 Sydney School of Public Health, University of Sydney, Camperdown, Australia; 4 Centre for Behaviour Change, University College London, London, UK; 5 Dept of Public Health, Environments and Society, Dept of Population Health, London School of Hygiene and Tropical Medicine, London, UK; 6 International Institute for Applied Systems Analysis (IIASA), Air Quality & Greenhouse Gases (AIR), Luxemburg, Austria; 7 African Population and Health Research Center, Nairobi, Kenya; 8 Clinical, Educational and Health Psychology, Division of Psychology and Language Sciences, University College London, London, UK; 9 Institute for Global Health, University College London, London, UK; 10 Tampere University, Tampere, Finland; 11 Bartlett School of Planning, University College London, London, UK

**Keywords:** City transformation; Sustainable urban development, Population health, Environmental policy, Public engagement, Participatory research

## Abstract

This paper describes a global research programme on the complex systemic connections between urban development and health. Through transdisciplinary methods the *Complex Urban Systems for Sustainability and Health* (CUSSH) project will develop critical evidence on how to achieve the far-reaching transformation of cities needed to address vital environmental imperatives for planetary health in the 21st Century. CUSSH’s core components include: (i) a review of evidence on the effects of climate actions (both mitigation and adaptation) and factors influencing their implementation in urban settings; (ii) the development and application of methods for tracking the progress of cities towards sustainability and health goals; (iii) the development and application of models to assess the impact on population health, health inequalities, socio-economic development and environmental parameters of urban development strategies, in order to support policy decisions; (iv) iterative in-depth engagements with stakeholders in partner cities in low-, middle- and high-income settings, using systems-based participatory methods, to test and support the implementation of the transformative changes needed to meet local and global health and sustainability objectives; (v) a programme of public engagement and capacity building. Through these steps, the programme will provide transferable evidence on how to accelerate actions essential to achieving population-level health and global climate goals through, amongst others, changing cities’ energy provision, transport infrastructure, green infrastructure, air quality, waste management and housing.

## Disclaimer

The views expressed in this article are those of the author(s). Publication in Wellcome Open Research does not imply endorsement by Wellcome.

## Background

By almost any objective measure, success to date in meeting key environmental and associated health challenges in cities around the world has, at best, been limited. Action to achieve increasingly urgent imperatives for planetary health has fallen far short of what is required. For example, most fossil fuel reserves must remain unburned to limit global heating to 2°C ( McGlade & Ekins, 2015; Pachauri *et al.*, 2014; UNEP, 2014). The Paris meeting of the UN Framework Convention on Climate Change in 2015 noted the importance of action for an even more stringent—and almost unachievable—target limit of 1.5°C above pre-industrial levels ( Rogelj *et al.*, 2016). At the same time, many urban populations still have inadequate access to improved water and sanitation or decent housing and will be vulnerable to extreme weather events.

The climate system is just one of nine planetary boundaries that are in danger of being transgressed, with serious implications for all countries ( Steffen *et al.*, 2015). There are, however, substantial potential benefits from climate action, not only in reducing future impacts of climate change, but also because of the more immediate ‘co-benefits’ for health of the transition to a low-carbon economy ( Haines *et al.*, 2009). For example, household and ambient air pollution contribute to millions of premature deaths ( GBD 2013 Risk Factors Collaborators, 2015) and their reduction is part of a climate change strategy.

There is abundant evidence that the future of health and natural systems in the Anthropocene will be determined by decisions on urban development ( Crane *et al.*, 2021). Population growth is focused in urban settlements which are responsible for a growing share of the world economy and greenhouse gas (GHG) emissions. Yet, opportunities to achieve benefits associated with policy and infrastructure investments are poorly understood and frequently overlooked. These are the focus of the *Complex Urban Systems for Sustainability and Health* (CUSSH) project.

## Aims and objectives

CUSSH is a 5-year Wellcome-funded research collaboration between six partner cities on three continents and 13 institutions. Working with our partner cities, the CUSSH project aims to conduct policy-relevant, actionable research to support the transformation of cities to meet environmental imperatives, including ambitious actions to decarbonize the economy, and to improve the health and wellbeing of current and future populations. It seeks to increase capacity for such transformations and to harness the benefits of sustainability-oriented policies, while minimizing the potential adverse consequences of global technological, environmental and social change. A key question is whether and how the use of scientific evidence, systems thinking and participatory engagement in decision processes can strengthen the planning and implementation of ambitious policies: this is our research agenda. CUSSH has five core objectives:

(1) To review potential solutions for healthy and sustainable urban development, which include technological innovations and changes to city governance, financing mechanisms and infrastructure, as well as human behaviour at individual, community and population levels;

(2) To establish methods for tracking and evaluating progress towards city-specific sustainability and health goals, and for comparing the impact of city development trajectories;

(3) To develop and apply a conceptual framework and models, to assess the impact of environmental policies on population health, health inequalities and socioeconomic and environmental parameters for various urban development pathways;

(4) To use systems-based, participatory and other research methods to undertake iterative engagements with stakeholders in the partner cities in order to evaluate and understand processes to help implement the transformative changes needed to meet local and global health and sustainability objectives;

(5) To develop a programme of public engagement and capacity building to ensure wide participation in the development (‘co-creation’) and use of research evidence by decision-makers and other stakeholders to help ensure environmental and health objectives receive appropriate weight in public policy.

Despite the challenge of connecting our broad objectives, the underlying logic relates to the development, role and application of scientific knowledge. Objectives 1, 2, and 3 are about rendering the scientific information useable and objectives 4 and 5 are about using it. A programme theory (see later section) describes how our objectives will be delivered and how we anticipate the programme will ‘work’ in practice.

## Underpinning principles

CUSSH is an international collaboration whose 13 institutions include teams from a diverse range of academic disciplines and non-academic fields. The six CUSSH city partners have different socio-political, geographical, environmental and city size contexts: Nairobi and Kisumu in Kenya, Beijing and Ningbo in China, and Rennes and London in Europe. Each geographical pair includes a capital and a smaller city and the result is a matrix of contrasting income levels, environmental challenges, and scale.

The research developed in partnership with the cities will encompass a range of city-specific topics. These include: (in Europe) research on the use of evidence on the impacts of principal environment and health policy initiatives for London and Rennes, methods to support regeneration of the Thamesmead area of south London, initiatives connected with urban investments in London; (in Kenya) research on spatial planning for health and sustainability in Kisumu and Homa Bay, the development of a waste management proposal for Kisumu to achieve health and greenhouse gas emissions reductions, and development planning for districts in Nairobi; and (in China) analysis of the measures and motives for change that have helped achieve recent air pollution improvements in China cities, analysis of the ancillary health effects of actions aimed at climate change mitigation, and planning of responses to heat risks in China cities.

Central to the CUSSH endeavour are the principles of *transformational change* and *transdisciplinary working*. The project seeks to support and enable change of a pace, scale and integration necessary to address pressing global challenges to environment and health. Such ambition requires fundamental transformative changes to the urban system and the physical, social and political structures, processes and values which underpin individual and collective behaviour ( Elmqvist *et al.*, 2019; Pelling *et al.*, 2015). Too often, urban sustainability or public health improvements are incremental, fragmented or aimed at achieving health or environmental outcomes in one small area, which limits the potential impact ( Crane *et al.*, 2021). Efforts to address climate change, for example, have often focused on individual infrastructure and technology interventions, such as developing solar panels for heating or electric vehicles for transport ( Heikkinen *et al.*, 2019). While such interventions could help reduce reliance on fossil fuels, neither alone addresses the broader issues of energy demand, the drivers of demand (beliefs, values and human behaviour) or energy use in the urban system as a whole. Opportunities to address urban sustainability challenges at the broader system-level can lead to improvements in health outcomes, and urban intervention should be considered via an integrated approach to both human and planetary health. The actions the CUSSH project aims to promote are based on multi-sectoral policies formulated by bringing together a wide range of actors, including policymakers, social and industry groups, researchers and community representatives ( Farla *et al.*, 2012; Köhler *et al.*, 2019), and which address city governance and policy implementation as well as urban planning and infrastructure development ( Smith *et al.*, 2005).

A second underpinning principle is that of *transdisciplinary working* ( Pineo *et al.*, 2021a), bringing together the knowledge, theories, and methods of a wide range of stakeholders. Stokols *et al.* (2013) define transdisciplinarity as “ *scholars and practitioners from both academic disciplines and non-academic fields working jointly to develop and use novel conceptual and methodological approaches that synthesize and extend discipline-specific perspectives, theories, methods, and translational strategies to yield innovative solutions to particular scientific and societal problems*”. Colleagues from CUSSH have built on the work of Stokols *et al.* (2013), to develop a new model ( Pineo *et al.*, 2021b) for transdisciplinary health research that entails (iterative) stages of co-learning, pre-development, reflection and refinement, conceptualisation, investigation and implementation. These stages are reflected in the framework of the project’s programme theory (see below). The practical translation of transdisciplinary working within the project is to encourage broad participation in team meetings and project governance to integrate diverse perspectives, to adopt participatory, behavioural science and social research methods, and to elicit knowledge from local communities and policymakers (e.g. see Dianati *et al.*, 2019 and Pineo *et al.*, 2021c)

## Research framework: a programme theory

The components of the project’s research and the evaluation of its impact are shaped by a programme theory elaborated through a participatory process of discussion among the wider consortium to ensure the input of a broad range of perspectives and shared understanding among team members ( Moore *et al.*, 2021). The programme theory is intended to explain how the project’s collaborative research will work to achieve its desired effects and how each of its various activities (tailored for each city) contributes to a chain of outputs that ultimately lead to change in the sustainability of the city and health of its residents ( Rogers, 2008; Stein & Valters, 2012). It also provides a framework for evaluation by guiding the evidence needed to assess (1) whether and how CUSSH achieves its aims for city health and sustainability, and (2) whether it improves transdisciplinary and cross-sectoral understanding and work.

**Figure 1.**
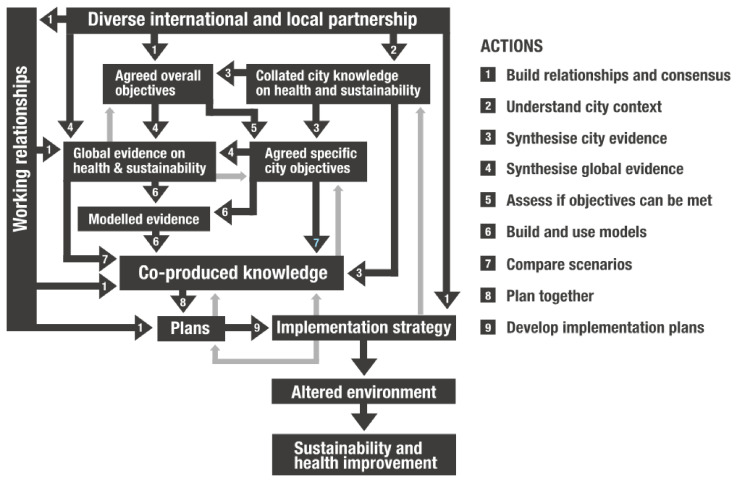
Action model for Complex Urban Systems for Sustainability and Health (‘CUSSH’). Dark arrows are actions. Light arrows are examples of feedback.

**Figure 2.**
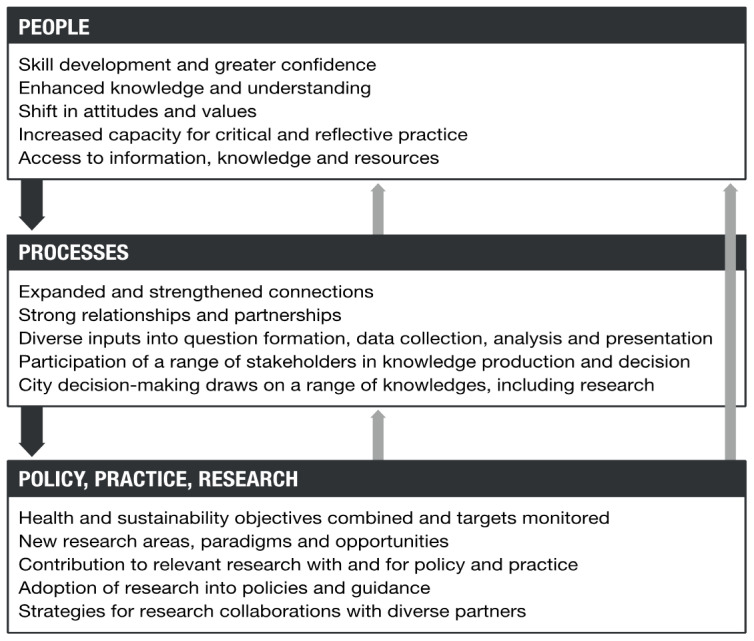
Change model for Complex Urban Systems for Sustainability and Health (‘CUSSH’). Dark arrows are feed-forward. Light arrows are feedback.

The programme theory has two elements: an ‘action model’ ( Figure 1) which describes the processes (boxes) and actions that are expected to achieve the steps of change (arrows), and a ‘change model’ ( Figure 2) which describes broad areas of change in people, processes, policies, practices and research.

The action model emphasises working relationships that lead to the co-production of knowledge used in developing participatory plans and implementation strategies that translate into improved city health and sustainability. The processes are likely to be non-linear and iterative. The change model emphasises the ways in which the programme might affect people, organisations and collectives. It recognises that participants in the research will develop their skills and may change the way they think about research and action. New ways of collaborating may develop from exposure to different disciplines, and the outcomes may be an example for other programmes.

The use of the programme theory to guide evaluation aims to ensure an integrative evaluation, including processes, outcomes, and (eventually) impacts. Evidence is collected through a variety of methods, including stakeholder surveys, document analysis, policy analysis, tracking and monitoring processes and indicators of change, qualitative interviews, and analytical memos. This will yield qualitative and quantitative data to generate an understanding of how the programme was implemented, its outputs and outcomes (where, how, and why they have occurred), as well as identifying unexpected positive or negative outcomes. CUSSH aims to contribute to changes in wider systems (i.e. governance, policy, research) and we recognise the challenges of operationalizing and evaluating ultimate changes. In Kisumu, Kenya, for example, alignment from the beginning with county government priorities and their involvement in the work on solid waste management and spatial planning might make sustained action more likely. We also acknowledge that the wider changes are not brought about by single interventions. From an evaluation point of view, any approach taken needs to be flexible in response to the open-ended nature of such outcomes, whilst also incorporating learning and refection. Our evaluation processes embodied in the action and change models allow both for the ongoing evaluation of the process of transdisciplinary work and evaluation of the (intermediate but not distal) outcomes. This paper describes the overall CUSSH programme in general: future publications will provide specific details of the evaluation of the programme.

## Methods of working

The project is based on (1) the generation of evidence about the impact of environment and health actions, and (2) participatory engagements between the research team and city stakeholders to share understanding and help shape programme and policy development and implementation.

### Evidence generation

Evidence generation has three components:

(i) The assembly of evidence from published literature on challenges and associated interventions for urban health and sustainability as a resource to help inform policy development. This includes (1) a literature review of healthy sustainable urban development and the factors that promote or impede its realisation, brought together as a concepts review; (2) the assembly and analysis of a global database of published peer-reviewed studies on implemented city interventions for climate change mitigation and adaptation relevant to health and wellbeing, to further examine specific questions relating to the impact and effectiveness of potential solutions, including those relating to behaviour change, infrastructure development and technological innovation, as well as exploring factors that have influenced the implementation of such solutions; and (3) the development of a classification of urban interventions for sustainability and health which will be analysed with respect to their potential impact at population scale (city level) on both GHG emissions and health outcomes.

(ii) The assembly of data to track progress towards achieving selected city-specific sustainability and health goals (consistent with global and local environment and health priorities) and also to draw lessons about the opportunities for healthier, more sustainable, development from trajectories of cities in different settings. Indicators track progress on environmental exposures and their associated health impacts and are largely based on secondary analysis of existing data sources. Where possible, these data will be compiled to show time trends over years to assess the context of recent changes and with acquisition of data for selected other similar cities as comparison. The core suite of indicators is intended to include measures of GHG emissions, ambient particle pollution (PM _2.5_) and meteorological data. More specific indicators match the foci of work in each city. So, for example, in London, where there is a specific focus on green infrastructure in the Thamesmead area, we are assembling indicators on access to and use of green space, while in Kisumu we are developing indicators relating to waste management to track the changes associated with proposals for improved municipal solid waste disposal and biogas facilities. Where possible, we will analyse the change in indicators against a trajectory of intended improvement and use modelling of associated health impacts to assess the degree to which health benefits are or are not realised through successful implementation of agreed policies. These data will be an important input to discussions with cities on assessing the speed of change against agreed targets.

(iii) The development and application of models to generate evidence on the effects of specific policies on human health and sustainability in the target cities. This includes the analysis of health-related behaviours and exposures, GHG emissions and health impacts. They include models of active transport, implementations of the ‘Greenhouse Gas – Air Pollution Interactions and Synergies’ (GAINS) model, microsimulation and System Dynamics models, which are deployed as appropriate to the specific questions in each setting.

In addition, a simplified tool, ‘Cities Rapid Assessment Framework for Transformation’ (CRAFT), is designed for the rapid comparison of policy options in terms of health and greenhouse gas emissions ( Symonds *et al.*, 2020). The model is based on comparatively simple assumptions and methods but is intended to allow the rapid comparison of different policy options before more detailed modelling.

Owing to the large differences between CUSSH cities, the granularity of the modelling necessarily differs between settings. In the Kenyan cities in particular, the limited availability of data poses quite strong constraints. With its flexible approach, CUSSH aims to strike a balance between data-driven detailed modelling and simpler calculations which can still inform the directions and magnitudes of expected effects from individual policies.

## Systems thinking and participatory engagement with cities

Participatory engagement with cities is a core activity of CUSSH research. It is the iterative process that allows the co-creation of research, the open exchange of ideas among the research team and city stakeholders, the consideration of research evidence (generated by the activities described under 5.1) and the co-development of policy ideas. The engagement is based on workshops and other meetings, usually involving a wide range of stakeholders.

The CUSSH project arose from the understanding that cities are complex systems ( Rydin *et al.*, 2012; Tan *et al.*, 2019), characterised by diverse priorities, mutual interdependences, feedback relationships and inherent delays, making it difficult for decision-makers to anticipate the consequences of their actions ( Richardson, 2011). Building on the team’s preceding research ( de Gooyert *et al.*, 2020; Dianati *et al.*, 2019; Eker *et al.*, 2018; Macmillan *et al.*, 2016; Pluchinotta *et al.*, 2021; Shrubsole *et al.*, 2019; Zimmermann *et al.*, 2020), the project takes a systems approach to address this complexity.

The process entails clarifying the issues that need to be addressed, investigating their causes, co-developing solutions and supporting implementation, informed by behavioural and implementation science. While the approach is based on simple steps, adopting a systems perspective may reduce unintended consequences by avoiding the common pitfall of jumping to solutions without having generated a joined-up understanding of the issues and their potential causes ( Dwyer & Stave, 2008). We incorporate qualitative and quantitative system dynamics modelling for policy analysis and design to help understand the feedback-rich system structure ( Sterman, 2000).

This structure includes local stakeholder priorities, infrastructure, decision-making processes and relationships, informed by an understanding of human behaviour, that have influenced sustainability and health outcomes in cities in the past and that we will need to successfully change for positive outcomes in the future. The approach recognises that city-wide transformation is not possible without people (policymakers, planners, the public) changing their mental models and behaviour. Enabling and setting up systems to support this is not easy, but there is a science of behaviour and behaviour change that CUSSH draws upon. For behaviour to change, there needs to be not only capability (knowledge and skills), but also motivation and the opportunity, physical and social, for behaviour to change. This is represented by the Capability, Opportunity and Motivation (COM-B) model which acts as a guiding framework; by understanding behaviour in its context, one can identify interventions and policies most likely to be effective ( Michie *et al.*, 2011).

## Public engagement

Public engagement is central to the CUSSH programme, to (1) increase the quantity and quality of public discussion—local and national—of research findings and the broader issues of urban health and environmental sustainability, and (2) help examine pathways through which publics—urban residents, artists, media, community and non-government groups—can use data to influence policy development in local, culturally diverse contexts.

Informal settlements in one of the six CUSSH partner cities, Kisumu, Kenya, will be a key urban space for examining engagement on a pressing issue for human and environmental health, identified by local partners.

Our objective is to use this part of the programme to understand how we might bring the public into decision-making. Three dimensions of the programme are designed to help with this: the practice of participatory system dynamics with decision-makers to develop a model for collaborative planning, particularly in terms of the translation of technical evidence; the involvement of county government in the community engagement activities in order to encourage two-way communication of issues and potential solutions; and the attempt to generate ‘heft’ by increasing the profile of the issues and local responses through media and the website. Implicit in this is the idea that increasing the profile of community voices will push decision-makers to listen.

The initial focus is on community management of solid waste, a pressing issue for which an analytical investigation by CUSSH showed how the actors involved hold different, often contrasting views about the sources of the underpinning causes and possible solutions ( Salvia *et al.*, 2021). Public engagement may contribute towards the alignment or at least the integration of divergent stakeholder views, in order to limit chances of unintended consequences from policy implementations. Residents will participate in a comprehensive and inclusive outreach programme involving 60,000 households. Engagement will include community dialogue and participatory local action workshops, activities such as data-gathering walks and social mapping, interaction with artists, film and radio co-production, and media training for local youth and journalists.

We aim to stimulate an increase in the quantity and quality of public discussion of sustainability and health in Kisumu, evidenced by both local action and media coverage. We will evaluate these by collating reports in local and national media, and assessing changes in confidence, output, and communication between local residents, journalists, researchers, and decision-makers. We will conduct qualitative interviews with residents and documentation of local initiatives through film and photography, with a particular interest in the influence on County Government policy of solutions generated by citizens.

The work will generate a range of products, including visual, audio, and text materials documenting activities and solutions developed through participatory processes facilitated by Kenyan researchers and creatives (in partnership with county government). These will be showcased for local and global audiences and decision-makers through a project website, with two purposes: to validate and increase the profile of community-led approaches to urban health and sustainability by helping to make them visible to individuals, decision-makers, and the wider world, and to encourage the involvement of municipal decision-makers in dialogue (through positive incentives for inclusion and negative incentives for exclusion).

## COVID-19

Within CUSSH we are liaising with our partner cities to restructure the programme where possible in order to address issues raised by the COVID-19 pandemic. The global response to COVID-19 has shown that rapid large-scale behavioural changes in societies are possible. Some of these changes, though not all, have pronounced environmental benefits. Examples include the increased action around the world to promote active travel (walking and cycling) and the demonstration, through encouraged and enforced remote working, of the benefits of reducing the need to travel ( Sung *et al*, 2020; Mohajeri *et al*, 2021). The pandemic has also exacerbated existing social divisions and inequalities in most countries. It is not yet clear, however, whether the positive changes will be maintained. For example, the observed reductions in ambient air pollution which are likely only to be mostly temporary ( Kumar *et al.*, 2020; Le Quéré *et al.*, 2020; Mohajeri *et al.*, 2021). There have been widespread calls for a ‘green’ recovery from COVID-19 that integrates action to improve health, equity, as well as environmental and economic objectives ( Guerriero *et al.*, 2020) with the aim of ‘Building back better’ (e.g. OECD, 2020). Cities will be critical to achieving such a ‘green’ recovery and there is an opportunity for the CUSSH programme to interact with and influence their post-COVID agendas.

COVID-19 has had an unexpected and disruptive influence with substantial bearing on the CUSSH project. The pandemic has altered the ability of cities and research partners to contribute to some of the original CUSSH objectives and has also altered the policy priorities of many cities. Not only have cities had to turn their attention to the urgent measures to respond to and control the spreading of COVID-19, but they have also begun to re-evaluate policy opportunities and objectives given the very different post-COVID-19 context. This has led to requests from cities to the research team to contribute to new policy questions and evaluations. At the same time, the research team has chosen to introduce new elements of research that address COVID-19-related questions. 

## The future

Our ambition in the CUSSH project is to develop evidence on the connections between urban health and environmental sustainability to help accelerate transformative actions. To generate this evidence we are developing new, integrated modelling methods and ways of engaging with stakeholders via a framework which recognises the complex systems nature of cities. The aspiration is to use such improved knowledge to accelerate action at scale and pace on both local and global priorities. Our programme theory sets out what actions we will take and where we expect to contribute to change. We will use the programme theory as the basis of a detailed evaluation of the CUSSH approach. We hope that our work will inform an urgently needed new global model of action-oriented research via a much larger network of cities designing, implementing, testing and refining city-scale strategies.

## Data availability

No data are associated with this article.
