# Supplementary material for: The CUSSH programme: supporting cities’ transformational change towards health and sustainability
Source: Wellcome Open Res. 2021 Nov 9;6:100. Originally published 2021 May 7. [Version 2] doi: 10.12688/wellcomeopenres.16678.2 (PMC8686329; doi:10.12688/wellcomeopenres.16678.2)
Supplement: Peer review history

# Peer review: Reviewer response for version 2

**DOI:** 10.21956/wellcomeopenres.19186.r47008

**Published:** 2021-12-17

**Authors:** Joannette J. Bos, David Robertson

**Affiliations:** 1 Monash Sustainable Development Institute, Monash University, Clayton, Vic, Australia; 2 Monash Sustainable Development Institute, Clayton, Vic, Australia

My co-reviewer and I have had a look at this and are happy to upgrade it to an approval. I will provide some high-level comments below:

 Overall the revised version has improved its clarity, eg:

- Much clearer description of the target cities, timeframe, and examples of kinds of projects/programs.
- Useful summary point on the relationship of scientific information being 'useable' and 'used' between the major objectives (1/2/3 and 4/5).
- Clearer explanation of the transdisciplinary learning framework, though still a bit thin on how it looks in practice.
- Clearer explanation of the role of evaluation and potential challenges.
- Slightly clearer on COM-B and behaviour - this is being used to influence others, not as a self-examination lens.
- The public engagement artefacts are turned into 'attractive knowledge products' and this made it a lot more palatable and practical.

Does the article adequately reference differing views and opinions?

Partly

Are all factual statements correct, and are statements and arguments made adequately supported by citations?

Partly

Is the Open Letter written in accessible language?

Yes

Where applicable, are recommendations and next steps explained clearly for others to follow?

No

Is the rationale for the Open Letter provided in sufficient detail?

No

Reviewer Expertise:

The combined expertise of the reviewers is: participatory engagement, governance experimentation, learning processes, science communication, transdisciplinary and urban water and sustainability.

We confirm that we have read this submission and believe that we have an appropriate level of expertise to confirm that it is of an acceptable scientific standard.

# Peer review: Reviewer response for version 1

**DOI:** 10.21956/wellcomeopenres.18391.r44584

**Published:** 2021-07-29

**Authors:** Joannette J. Bos, David Robertson

**Affiliations:** 1 Monash Sustainable Development Institute, Monash University, Clayton, Vic, Australia; 2 Monash Sustainable Development Institute, Clayton, Vic, Australia

This open letter articulates the objectives, action model, change model and key areas of the CUSSH program. While the abstract is not completely clear about what the open letter seeks to achieve, the program described in the paper addresses important questions and seeks to improve capacity and implementation of initiatives to support sustainable city transformation.

 We have three thematic areas where we feel the open letter could be improved to better communicate its purpose, key project details and processes to support readers in understanding the value and areas of novelty of CUSSH. These are to provide a clearer explanation of project structure and scope; to further unpack the role of learning in the programme; and explaining how the program’s technical and participatory components and processes will interact in practice.

**Clarity on project structure and scope:**

 Structurally, this article could be greatly strengthened by articulating more explicit project parameters, such as timelines, the number, selection and locations of the partner cities, and at least a brief discussion of how geographic differences are to be handled in the action and change models. While CUSSH objectives and principles are made clear, these feel intangible without clarity on real-world project parameters. We were forced to seek this information via the CUSSH website while reading the open letter to make sense of certain statements, such as in the evidence section, that there may be limited data availability in “the Kenyan cities in particular.” This information would support readers to interpret and evaluate the programme theory; for example, the ‘Change Model’ (Figure 2) may require considerable tailoring of approach when evaluating “people” and “processes” between, for example, London, Beijing and Kisumu. 

 The “Action Model” figure offers some clarity about the actions and their relationships. We were surprised to note that it indicates CUSSH will not monitor, evaluate or create feedback loops beyond the implementation strategy phase - i.e, whether the “altered environment” and “sustainability and health improvement” are actually achieved in practice. Objective 4 seeks to “evaluate and understand processes to help implement transformative changes” - is this focused on developmental evaluation of the process of transdisciplinary work and planning; or is evaluation of outcomes (post-implementation) also included? If not the latter, to what extent does this undermine the overall value of insights from CUSSH, as it does not appear to ‘close the loop’ between objective 4 and objective 1?

**“Learning” in focus:**

 As reviewers, we felt there was an unresolved tension in this paper. “Learning” is present in the title as a key verb, and in the Change Model, explicit mention is given to skill development, enhanced knowledge, and increased capacity. However, we were unclear whether CUSSH has a clear learning agenda. Deliberate learning activities including reflection do not seem to be intentionally integrated into project activities in the Action Model. In the paragraph accompanying Figure 2, it is claimed that the project “might” or “may” change the way participants think and act - is this expected to be an emergent outcome of other project activities, or will there be interventions and actions that deliberately develop and evaluate such learning?

 In “Evidence Generation” (ii) point 2, brief mention is made of “evaluation of the influence of CUSSH initiatives with decision-makers” and, in the opinion of the reviewers, this could potentially generate more novel and transferable insights than some of the other evidence generation activities in this section, such as the collection of meteorological data - the latter of which are given more explanation. Greater clarity around the mechanisms and approaches used to deliver and evaluate features in the change model would benefit this open letter.

**Balance of technical contribution and participatory engagement:**

 We are encouraged to see considerable space dedicated to participatory and public engagement in the open letter. However, the section on participatory engagement is stated to be a “core activity”, but detail on the actual implementation and evaluation of this remains thin. We would like the open letter to be clearer on how tools such as COM-B will be used. Will this be targeted at participants (such as decision makers and researchers) to better enable the Action and Change Models, or is this expected to be deployed ‘externally’, for implementation of sustainability and health initiatives?

 We found the articulation of the public engagement program to be implemented in Kisumu exciting and potentially capable of generating interesting insights and outcomes. However, we have two major unresolved questions about this aspect of CUSSH which could be more clearly articulated in the open letter. The first is the relationship of this program with the earlier-described action model. Where does this fit, if it is expected to integrate with other activities at all? For example, will public engagement meaningfully influence the “agreed objectives” in this city - and not in other cities? Secondly, some of the language associated with the evaluation and outcomes of the program concerned us. “[S]howcasing” of public engagement outcomes as “attractive, intelligible knowledge products” on a “bespoke website” as a “model for public engagement elsewhere” implies a conceptual separation of the public engagement program (and its anticipated outcomes) from more technical evidence and modelling to decision-makers, as articulated by the Action Model. Without clarity on details such as time-frames, it is hard to know whether the public engagement activities are likely to meaningfully interact with more technical evidence or implementation planning processes explained earlier.

**Summary:**

 This open letter culminates in an impression for us, as reviewers, that the core logic informing the Action Model of CUSSH is that of a ‘deficit’ model. Objective 5 and the Change Model indicate that CUSSH seeks to create conditions for co-creation of evidence to support transformational change and transdisciplinary work. However, objectives 1, 2 and 3 are placed first and phrased in a way that does not imply inclusive, open process. We acknowledge that, in a short open letter, such a complex project can be difficult to articulate. There are multiple short mentions of promising principles such as nonlinearity and iterative feedback. We believe the open letter would substantially benefit by more clearly explaining how objectives 4 and 5 connect to, frame and interact with objectives 1, 2 and 3, as we feel there is significant potential for CUSSH to generate new insights in these domains.

Does the article adequately reference differing views and opinions?

Partly

Are all factual statements correct, and are statements and arguments made adequately supported by citations?

Partly

Is the Open Letter written in accessible language?

Yes

Where applicable, are recommendations and next steps explained clearly for others to follow?

No

Is the rationale for the Open Letter provided in sufficient detail?

No

Reviewer Expertise:

The combined expertise of the reviewers is: participatory engagement, governance experimentation, learning processes, science communication, transdisciplinary and urban water and sustainability.

We confirm that we have read this submission and believe that we have an appropriate level of expertise to confirm that it is of an acceptable scientific standard, however we have significant reservations, as outlined above.

# Response

**Published:** 2021-11-01

**Authors:** Nahid Mohajeri

**Affiliations:** UCL, UK

**Revision Notes**

 The CUSSH programme: supporting cities’ transformational change towards health and sustainability

**We would like to thank the reviewers for their very constructive and helpful comments, which we have carefully considered (and mostly followed) during the revision of the paper. We provide a new version of the paper. The version is marked as *‘Changes shown’*, where all changes made in response to the suggestions of the reviewers are shown in red. Below, we outline our response to each of the reviewers’ comments. In addition to these main changes, in response to the comments by the reviewer, we have made textual improvements, editing/formatting the references and clarifications throughout the text.**

**A copy of this response to the reviewers presented in a table format can also be viewed here.**

**Reviewer Report**

 Joannette J. Bos, Monash Sustainable Development Institute, Monash University, Clayton, Vic, Australia 

 David Robertson, Monash Sustainable Development Institute, Clayton, Vic, Australia 

 Approved With Reservations

**Reviewer comments:**

- The abstract is not completely clear about what the open letter seeks to achieve

**Response to the comments:**

- We have added some text to the abstract and elsewhere to clarify that the purpose of this paper is simply to describe the CUSSH programme (rather than also address in depth issues relating to the evaluation of the programme, for example).

**Reference to the text:**

- Sections: (i) Abstract (ii) Research framework: a programme theory

***Clarity on project structure and scope:***

**Reviewer comments:**

 1. While CUSSH objectives and principles are made clear, these feel intangible without clarity on real-world project parameters

**Response to the comments:**

- We have added some specific information about the cities we are working with.

**Reference to the text:**

- Sections: (i) Aims and objectives (ii) Underpinning principles

**Reviewer comments:**

 2. This information would support readers to interpret and evaluate the programme theory; for example, the ‘Change Model’ (Figure 2) may require considerable tailoring of approach when evaluating “people” and “processes” between, for example, London, Beijing, and Kisumu.

**Response to the comments:**

- The programme theory (detailed in a separate paper) describes our intentions at a programme level. The theory outlines the logic of the programme, specifically how we think our activities will “work” and how they will lead to changes. We agree that tailoring will be required and have now noted this in the paper. We note though that the development of our programme theory used examples from the cities – these were crucial to inform our thinking and our narrative of the CUSSH programme.

**Reference to the text:**

- Section: Research framework: a programme theory

**Reviewer comments:**

 3. Objective 4 seeks to “evaluate and understand processes to help implement transformative changes” - is this focused on developmental evaluation of the process of transdisciplinary work and planning; or is evaluation of outcomes (post-implementation) also included? If not the latter, to what extent does this undermine the overall value of insights from CUSSH, as it does not appear to ‘close the loop’ between objective 4 and objective 1?

**Response to the comments:**

- In recognition of the timeline of city level policy development and implementation, we acknowledge that it is not possible for us to monitor ultimate outcomes within the lifetime of the original programme. However, the evaluation processes embodied in the action and change models allow for both ongoing evaluation of the process of transdisciplinary work and evaluation of the (intermediate but not distal) outcomes. We have added some text to the paper to note this explicitly.
- Objective 4 in full states “t *o use systems-based, participatory and other research methods to undertake iterative engagements with stakeholders in the partner cities in order to evaluate and understand processes to help implement the transformative changes needed to meet local and global health and sustainability objectives*”. Objective 4 therefore focuses on the processes to achieve change – the ‘how’. Whereas objective 1 is about the ‘what’.

**Reference to the text:**

- Section: Research framework: a programme theory

***“Learning” in focus:***

**Reviewer comments:**

 4. However, we were unclear whether CUSSH has a clear learning agenda.

**Response to the comments:**

- Thank you for pointing this out. The intention of this paper isn’t to describe the learning or the learning process of CUSSH, but to describe the overall CUSSH programme. As stated above, we have now noted this in the abstract and provided information about the cities. We have also removed the word ‘learning’ from the title. (Please also see our response to point 5 below).

**Reference to the text:**

- Title

**Reviewer comments:**

 5. Deliberate learning activities including reflection do not seem to be intentionally integrated into project activities in the Action Model. In the paragraph accompanying Figure 2, it is claimed that the project “might” or “may” change the way participants think and act - is this expected to be an emergent outcome of other project activities, or will there be interventions and actions that deliberately develop and evaluate such learning?

**Response to the comments:**

- We have an evaluation plan which is tied to the programme theory, and this has a strong focus on reflection.  The evaluation plan (which will be published separately), isn’t within the scope of this brief paper which simply describes the CUSSH programme. As noted above, this has now been clarified in the abstract.
- In answer to the latter question, we think that the changes described by the reviewers are emergent. The original plan wasn’t really along these lines, but more positivist or instrumental. We do, however, have interventions to develop the learning, such as workshops on engagement.

**Reference to the text:**

- Abstract

**Reviewer comments:**

 6. In “Evidence Generation” (ii) point 2, brief mention is made of “evaluation of the influence of CUSSH initiatives with decision-makers” and, in the opinion of the reviewers, this could potentially generate more novel and transferable insights than some of the other evidence generation activities in this section, such as the collection of meteorological data - the latter of which are given more explanation

**Response to the comments:**

- Thank you for pointing this out. On reflection, we think that this sentence doesn’t fit it in this section and so have removed it.  We intended ‘evidence generation’ here to refer more to the development of city indicators.

**Reference to the text:**

- Section: Evidence generation

**Reviewer comments:**

 7. Greater clarity around the mechanisms and approaches used to deliver and evaluate features in the change model would benefit this open letter.

**Response to the comments:**

 We have developed an evaluation framework against the programme theory and plan another paper which will focus on evaluation.  We did not include such detail in this brief paper which aims simply to describe the broad CUSSH programme.

**Reference to the text:**

- general

***Balance of technical contribution and participatory engagement***

**Reviewer comments:**

 8. We would like the open letter to be clearer on how tools such as COM-B will be used.

**Response to the comments:**

- We are using COM-B as a guiding framework around behaviour change rather than, as yet at least, explicitly applying it. We have clarified this in the text.

**Reference to the text:**

- general

**Reviewer comments:**

 9. The first is the relationship of this program with the earlier-described action model. Where does this fit, if it is expected to integrate with other activities at all? For example, will public engagement meaningfully influence the “agreed objectives” in this city - and not in other cities?

**Response to the comments:**

- The Kisumu public engagement activities aim to generate conversations at three levels: within communities, between communities and county government, and between communities and the media. Our goal is to understand processes through which communities can contribute to the development of plans and influence agreed objectives. How to do this is difficult to know. As the reviewers suggest, there is a risk that the energy invested in public engagement in Kisumu leads to a conceptual split. Our objective is to use this part of the programme to understand how we might bring the public into decision-making. Three dimensions of the programme are designed to help with this: the practice of participatory system dynamics with decision-makers to develop a model for collaborative planning, particularly in terms of the translation of technical evidence; the involvement of county government in the community engagement activities in order to encourage two-way communication of issues and potential solutions; and the attempt to generate ‘heft’ by increasing the profile of the issues and local responses through media and the website. Implicit in this is the idea that increasing the profile of community voices will push decision-makers to listen.
- We have added some text to the paper in relation to this.

**Reference to the text:**

- Section: Public engagement

**Reviewer comments:**

 10. Secondly, some of the language associated with the evaluation and outcomes of the program concerned us. “[S]howcasing” of public engagement outcomes as “attractive, intelligible knowledge products” on a “bespoke website” as a “model for public engagement elsewhere” implies a conceptual separation of the public engagement program (and its anticipated outcomes) from more technical evidence and modelling to decision-makers, as articulated by the Action Model.

**Response to the comments:**

- Apologies if our aim was not clear. We do not make a conceptual distinction between public engagement and the use of technical evidence. Technical evidence and the use of models will be incorporated in community activities as well as in discussions with decision-makers. Indeed, our aim was to make models available more widely, including on the web.
- The idea of showcasing public engagement outcomes in an attractive way has two purposes: to validate and increase the profile of community-led approaches to urban health and sustainability by helping to make them visible to individuals, decision-makers, and the wider world, and to encourage the visible involvement of municipal decision-makers in community activities.
- We have amended the text in question.

***Summary***

**Reviewer comments:**

 11. This open letter culminates in an impression for us, as reviewers, that the core logic informing the Action Model of CUSSH is that of a ‘deficit’ model.

**Response to the comments:**

- Within the programme there are different ways of approaching this – from linear approaches where researchers disseminate their work, to more relational based exchanges, to thinking about the role of the systems that need to be in place to support evidence use in practice in the field of sustainability and health.
- In short, whilst welcoming the debate, we would respectfully disagree that the logic behind the entire CUSSH programme is a deficit model. We find this rather too narrow in thinking about the programme. The underlying logic of CUSSH acknowledges it is more complex than what a deficit model suggests. We are striving for co-production of knowledge, as it is central to the action model.
- It seems to us that any disconnect between objectives 1, 2, and 3 and 4 and 5 is the result of *not* having the scientific consensus on what should be done. So 1, 2, and 3 are about rendering the scientific information useable and 4 and 5 are about using it.
- So, we do not propose to say anything about a deficit model in the paper but have edited the text to note the disconnect/challenges of connecting the objectives.

**Reference to the text:**

- Section: aims and objectives

**Reviewer comments:**

 12. We believe the open letter would substantially benefit by more clearly explaining how objectives 4 and 5 connect to, frame and interact with objectives 1, 2 and 3, as we feel there is significant potential for CUSSH to generate new insights in these domains.

**Response to the comments:**

- Please see our response to point 11 above.

# Peer review: Reviewer response for version 1

**DOI:** 10.21956/wellcomeopenres.18391.r44588

**Published:** 2021-06-18

**Authors:** Tollulah Oni

**Affiliations:** 1 MRC Epidemiology unit, University of Cambridge, Cambridge, UK

The paper clearly sets out the rationale for this action oriented, city-scale work and why there is need for urgent action that recognizes the complex systems in operation. The authors defined the underlying principles of transformational change and transdisciplinary action guiding the work, and the frameworks that guide how these will be operationalized.

 With respect to the impact of the pandemic, the authors note: “This has led to requests from cities to the research team to contribute to new policy questions or evaluations. At the same time, the research team have chosen to introduce new elements of research that address COVID-19-related questions.”

 If the authors already have this information to hand, it would be interesting to say more about what new policy evaluations have been requested and the new elements of research that have emerged.

Does the article adequately reference differing views and opinions?

Yes

Are all factual statements correct, and are statements and arguments made adequately supported by citations?

Yes

Is the Open Letter written in accessible language?

Yes

Where applicable, are recommendations and next steps explained clearly for others to follow?

Yes

Is the rationale for the Open Letter provided in sufficient detail?

Yes

Reviewer Expertise:

Public health and urban epidemiology.

I confirm that I have read this submission and believe that I have an appropriate level of expertise to confirm that it is of an acceptable scientific standard.
